# Microbiological Quality and Contamination Level of Water Sources in Isiolo County in Kenya

**DOI:** 10.1155/2018/2139867

**Published:** 2018-07-09

**Authors:** Abok Elisha Onyango, Michael Wandayi Okoth, Catherine Nkirote Kunyanga, Bernard Ochieng' Aliwa

**Affiliations:** Department of Food Science, Nutrition and Technology, University of Nairobi, P.O Box 29053–00625, Kangemi, Nairobi, Kenya

## Abstract

Water security and safety is of vital concern in arid and semiarid regions of Kenya. Potable water accessibility and supply is limited due to fluctuating climatic conditions and environmental pollution that lower the wholesomeness of most water sources. The aim of this study was to establish the suitability of these water sources for drinking and use in industrial food processing by the small and medium enterprises (SME's). The aim of this study was to establish suitability of these water sources for drinking and use in industrial food processing by the small and medium enterprises (SME's). A total of 60 surface and ground water sources samples were purposively collected aseptically from the four administrative units (Ngare Mara, LMD, Leparua, and Wabera) of Isiolo County. ISO 16649-3, 688-2, 7937, 9308-1, and 18744 were used for enumeration of* E.coli, Staphylococcus aureus*,* Clostridium pafringens*,* Coliforms*, and cysts. Highest mean* Clostridium pafringens* counts in ground and surface water were 1452 Cfu/ml and 3421 Cfu/ml, respectively. Mean* Staphylococcus aureus* counts were 740 Cfu/ml and 1333 Cfu/ml in surface water and ground water, respectively.* Escherichia coli* and* Coliforms* contamination accounted for 29.88 % and 88.2 %, respectively. Microbial counts in the water sources differed significantly (p≤0.05). Total coliforms had a significant negative relationship (r = -0.76) with residual chlorine. Ground and surface water sources were highly contaminated with microorganism to levels regarded as unsafe by the Kenyan and WHO standards for potable water. Point-of-use water disinfection is thus necessary.

## 1. Introduction

Water is an important component of every life [1–4]. Water supply and accessibility is goal 6 of the sustainable development goals (SDGs) and aims at ensuring environmental sustainability [3, 4]. Historically, efforts to ensure access to safe drinking and food processing water have been focused on the community based water sources [5, 6]. Most regions of the developing nations are experiencing shortage of potable water supply as improved water sources are only limited to urban areas [7]. Isiolo County has limited water sources that include both surface and ground water sources [8, 9]. In a bid to promote healthy living among inhabitants of the county, a reliable potable water access is essential for sustainable development, health, food production, and poverty alleviation [4, 10]. Water shortage and pollution of the readily accessible water sources are evident in many regions of the developing nations [3, 6, 11]. This is largely attributed to low level of personal hygiene and inadequate treatment facilities for water and wastes that are consequent pollutants [12].

Increase in population has exerted more pressure on the available water sources. Consequently, more than 1.2 billion people worldwide do not have access to safe water [13–15]. Millions of people die yearly from diarrheal disease and a larger proportion are children aged below 5 years [16]. Besides causing death, water-related diseases also prevent people from working and living active lives [17].

Water is susceptible to contamination with microorganisms and organic matter among other pollutants regardless of the source [3, 11, 18]. Significantly, microbial contaminants such as coliforms,* E.coli*,* Cryptosporidium parvum*, and* Giardia lamblia* compromise the safety of the water [19]. Presence of* Escherichia coli*,* Klebsiella, *and* Enterobacter *species in water is a likely indicator of the presence of pathogenic organisms such as* Clostridium pafringens*,* Salmonella, *and Protozoa [18]. These pathogens cause diarrhea, giardiasis, dysentery, and gastroenteritis, which is common among the rural dwellers of developing nations [2, 3, 8, 20–22].

In Isiolo County ground water is dominant over surface water and is less susceptible to bacterial pollution. The soil and rocks through which groundwater flows screen out most of the bacteria [23]. But freedom from bacterial pollution alone does not mean that the water is suitable for use in food processing and drinking. Many unseen dissolved mineral and organic constituents are present in ground water in various concentrations. Most are harmless or even beneficial; though occurring infrequently, others are harmful, and a few may be highly toxic [23]. There is need to establish the extent to which the ground and surface water used for drinking and food processing in Isiolo County are contaminated with microorganism. This shall then serve as a yardstick to adopt an effective water disinfection technology to supply potable water to the residents and mitigate the current prevalence of water borne illness.

## 2. Materials and Methods

### 2.1. Study Setting

The study was conducted in Isiolo County, Kenya. Isiolo is classified as arid and semiarid land (ASAL). For the purpose of this study, the sampling locations were distributed over the four administrative units of Leparua, Ngare Mara, LMD, and Wabera coded as 1, 2, 3, and 4, respectively. The water sources were then categorized as ground water sources, surface water sources, and chlorinated urban water sources. Purposive samples of each water source were drawn from the four administrative units.

### 2.2. Data Collection

Isiolo central was purposively selected for this study owing to its urban nature with diverse water sources as well as its accessibility compared to the other divisions. Purposive sampling was employed based on the available water sources in Isiolo central. The samples were first coded based on the type of water source as BH, SW, SPR, R, PAN, TROUGH, and RAIN representing borehole, shallow well, spring, river, pan, trough, and rain water, respectively. The second part of coding of 1, 2, 3, and 4 represented the administrative sampling locations of Leparua, Ngare Mara, LMD, and Wabera, respectively. The last part of the sample code consisted of alphabetical letters to represent the different sample sites of the same water source from the same administrative sampling location. As such BH2F is a code for borehole water (BH), sampled from Ngare Mara (2) and the sixth sample unit (site) of borehole water type from Ngare Mara (F). For chlorinated urban water, TAP was followed by a numerical number that was used for identification to represent the number of units since they are only available in one administrative sampling area and thus needed no administrative differentiation of the samples.

Water sampling was done as per APHA method [24]. The samples were transported to the laboratory for analysis within 48 hrs postsampling owing to the long distance between sampling points and analysis station.

### 2.3. Sample Size

Sixty water samples were purposively sampled aseptically for analysis from Isiolo central. The samples consisted of 35 and 20 ground and surface water sources samples, respectively, while 5 chlorinated urban water samples were collected at five different consumer tap points. Secondary data on total coliforms,* Escherichia coli*, and residual chlorine for treated Isiolo river water and raw water for over a period of 6 years (2011-2016) were collected from Isiolo Water and Sewerage Company (IWASCO). The secondary data was analyzed to establish the water quality trends prior to the study.

### 2.4. Analytical Methods

#### 2.4.1. Enumeration of* Escherichia coli*

Enumeration of* Escherichia coli* was done as described in ISO 16649-3 [25]. Purple colonies on the selective media typical of* Escherichia coli* were enumerated.

#### 2.4.2. Enumeration of Coagulase Positive* Staphylococcus aureus*

Colony counts of* Staphylococcus aureus* in water were enumerated as described by ISO 6888-2 [26]. Coagulase positive black colonies on the selective media typical of* Staphylococcus aureus* were enumerated.

#### 2.4.3. Enumeration of* Clostridium pafringens*

Colony counts of* Clostridium pafringens* were done as described in ISO 7937 [27].

#### 2.4.4. Determination of Total Coliforms

Enumeration of total coliforms was done as described by ISO 9308-1 [28]. All typical pink colonies on the selective media were enumerated.

#### 2.4.5. Enumeration of Cysts

Enumeration of cysts was done using microscopy techniques described by ISO 18743:2015 [29], ISO 18744.2016 [30] for Hookworm,* Cryptosporidium*, and* Giardia lamblia*, respectively. Microscopic morphological characteristics were used for the enumeration of Amoeba cyst.

### 2.5. Data Analysis

Analysis of variance (ANOVA) at 5 % level of significance to compare means of the microbial water quality among all the sampled surface, ground, and chlorinated urban water sources, using statistical analysis software (SAS) version 9.0. Least significant difference (Lsd) was used to separate the means. Significant differences were indicated by letters.

Pearson correlation was used to establish the relationship among the microbiological quality aspects of the sampled water as well as the secondary data at 5 % and 1 % levels of significance. The association between residual chlorine, coliforms, and* Escherichia coli* as obtained from the secondary data for raw and chlorinated Isiolo river surface water from IWASCO data base for 6 years preceding 2017 was done at 5 % level of significance.

## 3. Results

### 3.1. Mean Microbial Counts in Ground, Surface, and Chlorinated Urban Water Sources

The mean microbial counts for* Escherichia coli, *total coliforms*, Staphylococcus aureus*, and* Clostridium pafringens* in ground, surface, and chlorinated urban water sources for food processing and drinking in Isiolo County are shown in Table 1. Borehole and shallow well are the ground water sources. River, spring, rain, pan, and trough were the surface water sources. Chlorinated urban water was sampled from IWASCO consumer unit taps.

*Escherichia coli* was absent in trough water. Rain water had the highest mean counts for* Escherichia coli* contamination of 160 Cfu/ml.* Escherichia coli* contamination insignificantly differed (p≤0.05) among the water sources except for rain water.

Rain water had the least mean coliforms count of 170 Cfu/ml while shallow well had the highest mean coliform counts of 5185 Cfu/ml. Mean total coliforms counts across all the water sources differed significantly (p≤0.05).

Pan water had the least* Staphylococcus aureus* count of 8 Cfu/ml while rain water had the highest* Staphylococcus aureus* count of 2450 Cfu/ml. There were insignificant (p≤0.05) differences in the mean* Staphylococcus aureus* counts among the water sources.

Spring water had the highest mean* Clostridium pafringens* count of 8177 Cfu/ml whereas chlorinated urban water had the lowest mean* Clostridium pafringens* count of 131 Cfu/ml. There were significant differences (p≤0.05) among the water sources.

### 3.2. Parasitic Cysts in the Ground, Surface, and Chlorinated Urban Water Sources

All the water samples collected from surface, ground, and chlorinated urban water sources were analyzed for Amoeba cyst,* Giardia lamblia*,* Cryptosporidium oocysts*, and hook worm.

Spring water had one hook worm larvae per milliliter.* Giardia lamblia *cysts were found in open trough water. River water had one hook worm per milliliter. Generally, it was only the surface water sources that indicated parasitic cysts contamination. Cysts were absent in ground water and chlorinated urban water sources.

### 3.3. Mean Coliforms,* Staphylococcus aureus*, and* Clostridium pafringens* Counts in Ground Water

The mean* Clostridium pafringens*,* Staphylococcus aureus*, and Coliforms counts in ground water sources are shown in Table 2.

*Clostridium pafringens* was absent in 54.29 % of ground water samples. BH3E (sampling site 5, LMD borehole water) had the lowest* Clostridium pafringens* count of 125 Cfu/ml while BH1A (Sampling site 1, Leparua borehole) had the highest mean* Clostridium pafringens *count of 16500 Cfu/ml. The samples contaminated with* Clostridium pafringens* were mainly from Leparua and Ngare Mara administrative sampling areas which are remote areas far off the administrative Isiolo town. Significant mean difference (p≤0.05) occurred among* Clostridium pafringens* positive ground water samples. BH3F (sampling site 6, LMD borehole water) had the lowest coliforms count of 11 Cfu/ml while SW4B (sampling site 4, Wabera shallow well water) had the highest mean coliforms count of 27500 Cfu/ml. Coliforms were absent in 11. 8 % of the 35 ground water sources samples analyzed. Mean Coliforms counts significantly differed (p≤0.05) among the ground water source samples. Only 11.4 % of the ground water samples met the Kenyan standard requirement of zero coliforms counts for potable water. Majority of the ground water sources samples were contaminated with* Staphylococcus aureus*.* Staphylococcus aureus* was absent in only 35.29 % of the ground water sources. BH3D (sampling site 4, LMD borehole water) had the lowest mean* Staphylococcus aureus* count of 9 Cfu/ml while SW2H (sampling site 8, Ngare Mara shallow well water) had the highest mean count of 10600 Cfu/ml. Mean* Staphylococcus aureus* counts differed significantly (p≤0.05) among the ground water sources.

### 3.4. Mean* Escherichia coli* Counts in Ground Water Sources

The mean* Escherichia coli* counts in the ground water sources are presented in Table 3.* Escherichia coli* was present in only 22.9 % of the ground water samples. SW2A had the lowest mean* Escherichia coli* count of 9 Cfu/ml while BH2A had the highest mean* Escherichia coli* count of 205 Cfu/ml. The mean* Escherichia coli* counts significantly (p≤0.05) differed among the ground water sources.

### 3.5. *Escherichia coli*,* Staphylococcus aureus*, and* Clostridium pafringens* Contamination in Surface Water Sources

*Escherichia coli, Clostridium pafringens*, and* Staphylococcus aureus* counts in the surface water samples are as shown in Table 4.* Escherichia coli, Staphylococcus aureus*, and* Clostridium pafringens* mean values differed significantly (p≤0.05) among the positive surface water samples. Only 36.8 % of the 20 surface water samples tested negative for* Escherichia coli, Staphylococcus aureus*, and* Clostridium pafringens*. R1C had the lowest mean* Escherichia coli* count of 12 Cfu/ml while the highest* Escherichia coli* count was 165 Cfu/ml in R4A. SPR1A had the highest mean* Clostridium pafringens* count of 41500 Cfu/ml and the lowest mean* Clostridium pafringens *count was 22 Cfu/ml in SPR1C. The highest mean* Staphylococcus aureus *count was 3750 Cfu/ml in R3A whereas the lowest was 11 Cfu/ml in R4B.

### 3.6. Mean Coliforms Counts in Surface Water Sources

The mean Coliforms counts in the surface water sources are as shown in Table 5. Mean total coliforms significantly differed (p≤0.05) among the surface water sources. R1A had the lowest mean total coliforms count of 18 Cfu/ml while Trough2 had the highest mean Coliforms count of 27500 Cfu/ml. The coliforms counts in the surface water sources were higher than the recommended minimum limit of 0 Cfu/ml by the Kenyan standard for potable water.

### 3.7. *Escherichia coli*,* Clostridium pafringens*, Coliforms, and* Staphylococcus aureus* Counts Correlation in Surface Water Sources

The correlation among* Escherichia coli, Clostridium pafringens, *Coliforms, and* Staphylococcus aureus *in surface water sources is shown in Table 6.

*Staphylococcus aureus* had a significant positive relationship to* Clostridium pafringens* and* Escherichia coli *count (r = 0.52 and 0.472), respectively.* Clostridium pafringens, Staphylococcus aureus*, and* Escherichia coli* are all pathogenic and their occurrence is a likely indicator of primary contamination of the water sources with faecal matter. Total coliforms counts had a negative correlation to all the three pathogens.

### 3.8. Level of Microbial Contamination in Chlorinated Urban Water Sources

The mean microbial counts in chlorinated urban water supply at consumer unit taps are shown in Table 7.* Escherichia coli, Staphylococcus*, and* Clostridium pafringens *were absent in most of the chlorinated urban water sources. Only 40 % of the chlorinated urban water sampled at the consumer unit taps had* Clostridium pafringens*. Tap1 had 775 Cfu/ml while Tap2 had 12.5 Cfu/ml mean* Clostridium pafringens* counts which significantly differed (p≤0.05). Coliforms were present in 80 % of the chlorinated urban water sources. Tap2 had the highest mean coliforms count of 15000 Cfu/ml that significantly differed (p≤0.05) from those of Tap3, Tap4, and Tap5.

*Staphylococcus aureus* was present in 60 % of the chlorinated urban water samples. The mean* Staphylococcus aureus* counts insignificantly differed (p≤0.05) among the chlorinated urban water sources. The highest and lowest mean* Staphylococcus aureus* counts were 475 Cfu/ml and 75 Cfu/ml in Tap5 and Tap2, respectively. Similarly* Escherichia coli *was present in 40 % of the chlorinated urban water sources. Tap3 and Tap4 each had 13 Cfu/ml and 23 Cfu/ml of* Escherichia coli*, respectively. The mean value for* Escherichia coli* counts in the positive samples significantly differed (p≤0.05).

### 3.9. Level of* Escherichia coli* and* coliforms* Contamination in Raw Isiolo River Water

The daily analysis records for* Escherichia coli *and Coliforms for the raw unchlorinated Isiolo river water from Isiolo Water and Sewerage Company (IWASCO) were analyzed and the annual mean counts compared over the six years.  Table 8 shows the mean counts for* Escherichia coli* and total coliforms for the six years. The year 2013 had the highest Escherichia coli mean count of 807.1 Cfu/ml while the year 2012 had the lowest mean* Escherichia coli* count of 179.1 Cfu/ml. The highest mean coliform count was 1525 Cfu/ml in the year 2013 whereas the lowest was 340.2 Cfu/ml in the year 2015. Total coliforms and* Escherichia coli* significantly differed (p≤0.05) across the six years. Coliforms and* Escherichia coli* counts in Isiolo river are relatively similar to the counts observed in other surface river water sources shown in Tables 4 and 5. For raw Isiolo river water, the counts were higher since the data covers a wide range of daily fluctuations within a year which is not the case for the analyzed surface river water samples that were representative of the sampling day situation.

### 3.10. Level of* Escherichia coli* and* coliforms* Contamination in Chlorinated Isiolo River Water

The residual chlorine, coliforms, and* Escherichia coli* counts data for chlorinated Isiolo river water were obtained from the IWASCO daily analysis records. The daily data was computed for annual means and compared at 5 % level of significance over the 6 years. For chlorinated Isiolo river water. The highest mean* Escherichia coli* was 29.9 Cfu/ml in the year 2013 and the lowest was 5.6 Cfu/ml in the year 2014.* Escherichia coli* was absent in the tested water in 2011, 2015, and 2016. Little significant differences (p≤0.05) occurred in* Escherichia coli* counts across the years. The highest mean value for coliform was 54.6 Cfu/ml in the year 2013 whereas the lowest was 11.1 Cfu/ml in the year 2014. Total coliforms were absent in 2011, 2015, and 2016. Mean residual chlorine insignificantly (p≤0.05) differed. The highest mean residual chlorine was 0.439 in the year 2014 and the lowest was 0.395 in the year 2011.

### 3.11. Residual Chlorine,* Escherichia coli*, and Total coliforms Correlation in Chlorinated Isiolo River Water

The mean residual chlorine,* Escherichia coli*, and total coliforms correlation in chlorinated river Isiolo water are presented in Table 9.

Residual chlorine had a strong significant negative relationship to both* Escherichia coli* and total coliforms r = - 0.678 and - 0.766, respectively. An increase in* Escherichia coli* results in a significant (p ≤ 0.01) increase in total coliforms.* Escherichia coli* and total coliforms had a strong positive relationship (r = 0.893).

## 4. Discussion

### 4.1. Level of Coliforms,* Escherichia coli*,* Clostridium pafringens*, and* Staphylococcus aureus* in Ground Water Quality

Ground water is a major source of drinking water [31–33]. Its pollution by pathogens and elevated concentrations of dissolved solids is of concern due to its use for drinking and its effect on the quality of surface water bodies into which ground water discharges. Concentrations of total coliforms,* Escherichia coli, Clostridium pafringens*, and* Staphylococcus aureus* in the ground water samples were higher, indicating the extent of contamination of the water sources making them unsafe for food processing and drinking [31, 34].

The presence of* Escherichia coli*, total coliforms, and* Clostridium pafringens* in higher counts in ground water indicates contamination by potentially dangerous faecal matter and other pathogens that compromises the safety of such water sources [34]. Total coliforms presence in the water is therefore useful for monitoring the microbial quality of drinking water from time to time [11]. To minimize health risk resulting from the consumption of such contaminated ground water, appropriate treatment processes should therefore be utilized for disinfection of ground water for quality and safe food processing and drinking water [20]. Contamination of ground water by coliforms and* Escherichia coli* counts that exceed zero colony forming units per milliliter recommended for standard drinking water has been reported by Mahananda et al. [35] and Manhokwe et al. [36]. The level of microbiological contamination in the ground water exceeded the limits regarded as safe by East African standard for drinking water.

Groundwater sources are very important resource for drinking purpose because it has been found to contain over 90 % of the fresh water recharge over the world [36]. It is partially or severely polluted depending on the level of vulnerability to pollution sources. Poor microbiological quality of ground water sources is of concern at the point of use considering the health risk and the handling conditions at the household level where unhygienic practices dominates the handling operations [32]. There exists incessant microbial contamination of ground water among rural communities and Isiolo County is not an exception as shown by the level of microbial count [37, 38]. Consumption of contaminated ground water could therefore be a root cause of diarrheal conditions and deaths reported among the rural population of Isiolo County. Therefore, disinfection of ground water at the point of use for food processing and drinking is necessary.

### 4.2. Level of Coliforms,* Escherichia coli*,* Clostridium pafringens*, and* Staphylococcus aureus* in Surface Water Sources

Surface water covers a wide area of the Earth surface [39]. Springs, rivers, pans, and dams are the predominant surface waters in the rural areas of developing nations [6, 12]. These sources are susceptible to diverse contaminants given their open exposure to the environment [3, 6, 11]. The population of Isiolo County that uses these water sources for drinking and food processing are therefore exposed to higher health risk as shown by higher counts of coliforms,* Escherichia coli*,* Clostridium pafringens*, and* Staphylococcus aureus* that exceeded the recommended Kenyan standard for drinking water of zero colony forming units per milliliter [19].

*Escherichia coli* and coliforms presence in the surface water sources points out the possibility of contamination by other pathogenic microorganisms that further renders such water unsafe for drinking and food processing [18]. All the surface water sources samples tested positive for total coliforms. The counts exceeded the limits regarded as safe for drinking water by Kenyan standards. Such level of contamination exposes the end user community members to higher health risk and the prevalence of diarrheal conditions and other water borne infections in Isiolo can be explained by the continued use of contaminated surface water for drinking and food processing [32, 40].

### 4.3. Level of Microbial Contamination in the Chlorinated Urban Water Sources

About 80 % of the treated urban water supply samples tested positive for total coliforms. Similarly notable proportion of the samples tested positive for* Escherichia coli*,* Clostridium pafringens*, and* Staphylococcus aureus*. The mean values significantly differed (p≤0.05) indicating the recontamination at the consumer unit taps. Unhygienic handling practices at the consumer points of chlorinated water collection result in cross-contamination of the already disinfected water with pathogenic organism thereby compromising the safety of the water [40]. Inadequate sewerage system along the water supply chain and septic systems implicated leakages that pollute the water with pathogenic bacteria [32]. Some of the water samples tested negative for Coliforms,* Escherichia coli, Clostridium pafringens*, and* Staphylococcus aureus* indicating the adequacy of treatment post-chlorination disinfection recontamination. The tested chlorinated water samples and the analyzed secondary data for chlorinated Isiolo river data showed similar levels of contamination; this is attributed to higher initial load in the river water that partially withstand the disinfection effect induced by chlorine dose.

Despite the treatment given to the water, recontamination of treated water has been a trend of concern [18]. Lack of proper cleaning of the storage and handling containers has also been implicated in cross-contamination of water with pathogenic bacteria [41].

### 4.4. Mean Microbial Contamination of Water Sources in Isiolo County Kenya

Generally, surface water sources were more contaminated than the ground water sources. In most cases, surface water sources are contaminated by waste, sewage, and bacteria along the water flow paths. [3, 6, 11, 42]. The ground water passes through a bed of soil and rock as the surface water run-off infiltrates and percolates through the earth crust [40, 43]. Inappropriate tillage operations on arable lands on the slopes of Mount Kenya, the source of river Isiolo, Ayana river, and all the springs in Isiolo are major contributors to surface water pollution as similarly reported by Wang et al. [44]. In most cases the residents water their animals by driving them directly into the surface water sources. The animals urinate and defecates in the water. The animal's waste therefore forms the sources of faecal contamination noted by high levels of coliforms,* Escherichia coli*, and* Clostridium pafringens*. During sample collection, goats were observed grazing on riparian vegetation growing on the banks and surface of Ngare Ndare river, one of the surface water sources for most locals in Leparua area of Isiolo County. As the goats graze, they urinate and drop their faecal matter on the surface of Ngare Ndare river, hence the eventual observable contamination [40].

### 4.5. Parasitic Cysts Contamination Levels in Water Sources in Isiolo

Most of the water borne pathogens are zoonotic [40, 41].* Giardia lamblia, *Hookworm*, cryptosporidium*, and Amoeba are shed into the surface water sources from the skins of the animals as well as from the urine and faecal matter [42]. Inadequate hygiene facilities in most rural set-ups of Isiolo promote open human waste disposal [6]. The cysts of the gastrointestinal origin find their way into the surface soils. During precipitation the cysts are carried along in the surface run-offs to the open surface waters where they thrive given favourable environmental conditions [34]. Cysts contribute to malnutrition in children as they suck nutrients from the gastrointestinal tract of the host victims and their presence compromises the entire water safety [18].

### 4.6. Coliforms and* Escherichia coli* Contamination Levels in Raw Isiolo River Water

Total coliforms and* Escherichia coli* counts indicated that the Isiolo river water was not safe for direct use in food processing and drinking. High counts of coliforms in the river water were an indicator of the extent of pollution that points to the presence of other pathogenic bacteria [44]. Coliforms and* Escherichia coli* counts arise from defecated materials as well as direct discharge of sanitary wastes from the urban settlement through which the river pass. Isiolo County has limited hygiene facilities; this greatly contributes to faecal contamination of the river water [6]. In order to ensure safe water provision, disinfection at the use point is necessary [45]. Solar water disinfection can be an alternative to diversify on the boiling and chlorination disinfection methods [46].

### 4.7. *Escherichia coli*, Coliforms, and Residual Chlorine Levels in Chlorinated Isiolo River Water

The chlorinated Isiolo river water samples, analyzed in 2011, 2015, and 2016, tested negative for both* Escherichia coli* and total coliforms indicating the adequacy of the treatment thus meeting the safety requirements for potable water. However, the varied handling practices in 2012, 2013, and 2014 in some of the sampling points at consumers units recontaminated the already disinfected water. Therefore, creation of awareness and uplifting of personal hygiene standards at consumer level is necessary. Proper water handling facilities at point-of-use level should be adopted to minimize risk of cross-contamination along the water supply chain. Regular maintenance of the water supply pipes from IWASCO to the consumer's households and servicing the clogged pipes that provide habitat for bacterial regrowth would be a positive remedy [34]. Posttreatment regrowth of coliforms also occurs if the treatment was inadequate to completely inactivate and destroy them [3, 18, 47].

The mean residual chlorine levels in the chlorinated Isiolo river water were 0.417 ppm that was within the limit of less than 0.5 ppm recommended level for piped water [48]. This was adequate to maintain the coliforms level within the acceptable limits of zero colony forming units per milliliter. Variations in point-of-use handling practices resulted in significant counts of total coliforms in some of the water samples tested. Unhygienic handling of the water after chlorination may result in introduction of new colony forming units that grow and survive in the water lowering the microbiological quality further. But since microbial quality had a strong significant (r = 0.766) negative correlation with chlorine residual, increased chlorine dosage greatly lowers the microbial load to zero counts. This is effective to a limit of 0.5 ppm beyond which the chlorine dose renders the water unpalatable [44].

## 5. Conclusion

Surface, ground, and chlorinated urban water sources in Isiolo were contaminated with bacteria and cysts to levels regarded as unsafe as per the standards for potable water. This makes the water sources unsafe for direct drinking and use in food processing. Point-of-use water disinfection is needed. Solar water disinfection which uses clean and cheap solar energy to induce germicidal effect would be appropriate in the area owing to the high solar intensity of about 800 Wm_ _^−2^  in Isiolo. Acceptability of solar use in powering boreholes pumps and solar drying of agricultural produce has increased recently and solar water disinfection technologies might not be an exception. This would minimize health risk associated with other chemical disinfection methods and save on biofuel consumption in the form of firewood for boiling water as well.

## Figures and Tables

**Table 1:** The mean microbial counts for ground, surface and chlorinated water sources.

**Water source **	***E.coli (Cfu/ml)***	***Total coliforms (Cfu/ml)***	***Staphylococcus (Cfu/ml)***	***Clostridium (Cfu/ml)***
**Borehole**	13.91±9.16 a	2166±95.24ab	674±18.21a	1368±33.78a
**Spring **	35.92±8.89a	4955±29.92abc	895±24.14a	8177±29.55b
**Tap water**	6.0±0.54 a	2723±56.29abc	308±8.86a	131±13.92a
**Shallow well**	15.26±5.86a	5185±66.83abc	2183±25.47a	1020±22.16a
**Rain **	160.0±14.14b	170±14.14a	2450±12.13a	1500±27.11ab
**River **	42.59±7.21a	2079±48.59ab	448±45.95a	878±16.48a
**Trough **	0.0±0.0 a	14012±77.5 ac	1207±16.18a	3513±15.87ab
**Pan **	6.25±0.75 a	1635±88.21a	8.0±0.57a	1750±26.55ab

(1) Values are means of more than 10 determinations ± standard deviations.

(2) Values with the same letters on the same column are not significantly different at 5 % level of significance.

**Table 2:** Mean Coliforms, *Staphylococcus aureus*, and *Clostridium pafringens *in ground water sources.

**Water sources**	**Coliforms (Cfu/ml)**	**Water source**	***Staphylococcus aureus *(Cfu/ml)**	**Water source**	***Clostridium pafringens *(Cfu/ml)**
BH3F	11±1.4a	SW2E	4100±82.8c	SW2E	6950±33.6e
SW2E	25±7.1a	SW2N	3750±63.6c	SW2N	225±10.6a
SW2N	38±3.5a	SW2H	10600±84.8f	BH4A	1610±62.2bc
SW2H	52±1.8a	BH2G	12±2.8a	BH3C	165±49.5a
BH2G	152±24.7a	BH3C	405±21.2a	BH3E	125±35.4a
BH4A	180±28.3a	SW2L	275±35.4a	BH3B	400±14.1a
BH3C	190±14.4a	BH3D	9±1.4a	BH2C	585±13.4a
BH3E	245±21.2a	BH2A	15±1.7a	SW2B	2500±70.7cd
SW2L	255±35.4a	BH3B	58±3.5a	BH2B	2800±22.8d
BH3D	375±35.5a	BH2C	9600±56.5e	SW2A	6750±44.1e
BH2E	380±28.2a	SW2B	11±1.5a	BH1A	1065±62.6ab
BH2A	525±77.8a	SW2D	125±3.6a	SW2F	200±14.1a
BH3B	875±13.4ab	BH2D	280±28.2a	SW4B	2735±22.3d
BH2C	1195±27.6abc	SW2D	40±1.4a	**Mean**	**1452**
SW2B	1350±53.6abcd	SW2C	25±7.1a	**cv **%	**22.2**
BH2B	1500±28.3abcde	BH1A	28±3.5a		
BH1A	2200±34.67bcdef	SW2F	9500±70.7e		
SW2G	2250±33.6bcdef	BH4C	12±2.8a		
SW2A	2500±346.4cdef	SW2M	12±2.6a		
BH4D	2800±282.8cdef	SW4B	5400±84.9d		
BH2D	3000±28.2def	**Mean**	**1333**		
SW2D	3250±22.1ef	**cv **%	**20.0**		
BH3A	3500±70.7f				
SW2C	3750±53.6f				
BH1A	7250±49.97g				
SW2F	14500±77.1h				
BH4C	15250±106.1h				
SW2M	23500±121.3i				
SW4B	27500±335.5j				
**Mean **	**3562**				
**cv **%	**21.5**				

(1) Values are means of two determinations ± standard deviations

(2) Values with the same letters on the same column are not significantly different at 5 % level of significance.

**Table 3:** *Escherichia coli* counts in ground water sources.

**Water sample **	***Escherichia coli *(Cfu/ml)**
SW2A	9.0±1.3a
SW2N	12.5±2.1a
BH3E	14.0±3.6a
BH3C	17.5±2.8ab
SW2C	35.0±3.9bc
SW4A	50.0±4.7c
SW2G	135.0±8.2d
BH2A	205±10.1e

**Mean **	**59.34**
**Cv** %	**12.3**

(1) Values are means of two determinations ± standard deviations

(2) Values with the same letters on the same column are not significantly different at 5 % level of significance.

**Table 4:** *Mean Escherichia coli, Clostridium pafringens, and Staphylococcus aureus* counts in surface water sources.

**Sample **	***Escherichia coli (*Cfu/ml)**	**Sample**	***Clostridium pafringens *(Cfu/ml)**	**Sample**	***Staphylococcus aureus *(Cfu/ml)**
R1C	12.0±1.7a	SPR1C	22.0±2.3a	R4B	11.0±1.4a
PAN2B	12.5±2.9a	R3C	38.0±1.5a	PAN2A	15.0±1.6a
SPR3A	12.5±1.8a	R1A	125.0±3.7a	SPR1D	22.5±2.3a
SPR1D	13.0±1.6a	SPR1D	242.0±5.8a	SPR3A	25±3.4a
SPR1B	15.0±1.2a	R4B	338.0±10.1a	R4A	50.0±4.2a
R3C	35.0±1.3a	RAIN2	1500.0±15.5a	SPR1B	95.0±2.5a
SPR1A	40.0±2.5ab	R1C	1565.0±40.9a	TROUGH2	864.5±3.7b
SPR3B	55.0±1.4bc	R3A	1850.0±28.2a	TROUGH4	1550.0±10.8c
SPR1C	80.0±1.8c	PAN2A	3500.0±56.9a	SPR3B	1725±13.9c
R3A	145.0±5.3d	TROUGH2	7026.0±282.4b	RAIN2	2450.0±23.7d
RAIN2	160.0±12.5d	SPR1B	7300.0±457.8b	SPR1A	3500.0±45.9e
R4A	165.0±11.3d	SPR1A	41500.0±483.7c	R3A	3750±70.8e

**Mean **	**39.2**	**Mean**	**3421**	**Mean**	**740**
**cv **%	**23.3**	**cv **%	**46.1**	**cv **%	**26.5**

(1) Values are means of two determinations ± standard deviations

(2) Values with the same letters on the same column are not significantly different at 5 % level of significance.

**Table 5:** Mean total Coliforms in the surface water sources.

**Sample **	**Coliforms (Cfu/ml)**	**Sample**	**Coliforms (Cfu/ml)**
R1B	18±1.7a	SPR1C	975±13.5ab
R4B	28±2.1a	SPR3B	1200±11.3ab
R1C	38±2.8a	PAN2A	3150±17.2abc
SPR1B	95±4.6a	R4A	4100±15.4bc
SPR1A	110±3.6a	R3A	5500±25.6c
PAN2B	120±10.3a	R3C	5550±110.8c
RAIN2	170±12.5a	SPR3A	6350±282.2c
R3B	435±8.9ab	SPR1D	21000±53.6d
TROUGH4	525±16.8ab	TROUGH2	27500±83.7e
R1A	605±15.3ab		

**Mean **	**4077**	**Mean**	**4077**
**cv **%	**18**	**cv **%	**18**

(1) Values are means of two determinations ± standard deviations.

(2) Values with the same letters on the same column are not significantly different at 5 % level of significance.

**Table 6:** *Escherichia coli, Clostridium pafringens, Coliforms, and Staphylococcus aureus* counts correlation in surface water sources.

	***Staphylococcus aureus***	**Coliforms**	***Clostridium pafringens***	***E.coli***
*Staphylococcus aureus*	1	-0.053	0.52_ _^*∗∗*^	0.472_ _^*∗∗*^
Coliforms	-0.053	1	-0.032	-0.095
*Clostridium pafringens*	0.52_ _^*∗∗*^	-0.032	1	-0.018
*E. coli*	0.472_ _^*∗∗*^	-0.095	-0.018	1

^*∗∗*^Correlation is significant at the 0.01 level (2-tailed).

**Table 7:** Level of microbial contamination in chlorinated urban water sources.

**Sample **	***Coliforms (Log Cfu/ml)***	***Escherichia coli (Log Cfu/ml)***	***Clostridium pafringens (Log Cfu/ml)***	***Staphylococcus aureus (Log Cfu/ml)***
TAP5	200±4.9a	0.0±0.0a	0.0±0.0a	475±30.67b
TAP4	430±10.8a	23±0.67c	0.0±0.0a	0.0±0.0a
TAP3	710±23.6a	13±1.9b	0.0±0.0a	0.0±0.0a
TAP 1	0.0±0.0a	0.0±0.0a	775.0±59.5b	125.7±8.09b
TAP2	15000±45.3b	0.0±0.0a	12.5±1.71a	75.0±4.69b

**Mean **	**3268**	**7.2**	**157.5**	**135.14**
**cv **%	**19.5**	**16.7**	**10.7**	**6.4**

(1) Values are means of two determinations ± standard deviations.

(2) Values with the same letters on the same column are not significantly different at 5 % level of significance.

**Table 8:** Level of *Escherichia coli* and *coliform*s contamination in raw Isiolo river water.

**Year**	***Escherichia coli *(Cfu/ml)**	**Total coliforms (Cfu/ml)**
**2011**	262.4±36.38bd	1180±18.16e
**2012**	179.1±69.67bd	1287.6±64.79efg
**2013**	807.5±24.9e	1525.4±41.40eg
**2014**	665.5±45.6e	1227.5±66.91ef
**2015**	179.4±36.01bd	340.2±59.91bd
**2016**	285.1±28.68bd	613.6±12.16bd

(1) Values are means of > 10 determinations ± standard deviations.

(2) Values with the same letters on the same column are not significantly different at 5% level of significance.

**Table 9:** Association between Residual chlorine, *Escherichia coli*, and coliforms in chlorinated Isiolo river water.

	**Residual chlorine**	***Escherichia coli***	**Total coliforms**
Residual chlorine	1	-0.678_ _^*∗∗*^	-0.766_ _^*∗∗*^
*Escherichia coli*	-0.678_ _^*∗∗*^	1	0.893_ _^*∗∗*^
Total coliforms	-0.766_ _^*∗∗*^	0.893_ _^*∗∗*^	1

N= 677; ^*∗∗*^ correlation is significant at the 0.01 level (2-tailed).

## References

[1] Mberekpe P. B., Eze M. N. (2014). Effect of preservation on the quality of sachet water consumed by households in Nsukka zone. *International Institute for Science Technology and Education Journal*.

[2] Thliza L. A., Khan A. U., Dangora D. B., Yahaya A. (2015). Study of some bacterial load of some brands of sachet water sold in Ahmadu Bello University (Main Campus), Zaria, Nigeria. *International Journal of current Science*.

[3] Oludairo O., Aiyedun J. (2016). Contamination of commercially packaged sachet water and the public health implications: an overview. *Bangladesh Journal of veterinary medicine*.

[4] Edbert D. C., Sandra A. U., Ebere E. C. (2017). Storage and its Effect on Chemical Quality Indicators in Sachet Water Brands Sold in Owerri Municipal. *Journal of World News of Natural Sciences*.

[5] Fisher M. B., Williams A. R., Jalloh M. F. (2015). Microbiological and chemical quality of packaged sachet water and household stored drinking water in Freetown, Sierra Leone. *PLoS ONE*.

[6] Muhammad I., Anas F., Ahmad S. (2017). Determinants of Safe Drinking Water Supply in Nowshera District of Khyber Pakhtunkhwa. *American Journal of Water Resources*.

[7] Omalu I. C. J., Eze G. C., Olayemi I. K. (2010). Contamination of sachet water in Nigeria: assessment and health impact. *The Online Journal of Health and Allied Sciences*.

[8] Aroh K. N., Eze E. M., Ukaji D. (2013). Health and environmental components of sachet water consumption and trade in Aba and Port Harcourt, Nigeria. *Journal of Chemical Engineering and Materials Science*.

[9] Hamidu H., Lawal M., Abdulganiyu Y. (2017). Re-evaluation of Shallow Floodplain Aquifers Groundwater Potentials and Storage of Sokoto Basin, Northwestern Nigeria. *American Journal of Water Resources*.

[10] Balogun S. A., Akingbade A. O., Oyekunle M. A., Okerentugba P. O. (2014). Physiochemical and Microbiological profile of drinking water sold in Abeokuta, Ogun State. *Nigeria Nature and Science*.

[11] Gangil R., Tripathi R., Patyal A., Dutta P., Mathur K. N. (2013). Bacteriological evaluation of packaged bottled water sold at Jaipur city and its public health significance. *Veterinary World*.

[12] Kuta G., Emigilati M., Hassan A., Ibrahim I. (2014). Domestic water sources and its health implication in Lapai Local Government area, Niger State, Nigeria. *Ethiopian Journal of Environmental Studies and Management*.

[13] Boubetra A., Nestour F. L., Allaert C., Feinberg M. (2011). Validation of alternative methods for the analysis of drinking water and their application to Escherichia coli. *Applied and Environmental Microbiology*.

[14] Adegoke O. O., Bamigbowu E. O., Oni E. S., Ugbaja K. N. (2012). Microbiological examination of sachet water sold in Aba, Abia State, Nigeria. *Global Research Journal of Microbiology*.

[15] Maduka H., Chukwu N., Ugwu C. (2014). Assessment Of Commercial Bottled Table And Sachet Water Commonly Consumed In Federal University Of Technology, Owerri (FUTO), Imo State, Nigeria Using Microbiological Indices.. *IOSR Journal of Dental and Medical Sciences*.

[16] Muhammad M., Samira S., Farrukh J., Faryal A. (2013). Assessment of Drinking Water Quality and its Impact on Residents Health in Bahawalpur City. *International Journal of Humanities and Social Science*.

[17] Memon M., Soomro M. S., Akhtar M. S., Memon K. S. (2011). Drinking water quality assessment in Southern Sindh (Pakistan). *Environmental Modeling & Assessment*.

[18] Anyamene N. C., Ojiagu D. K. (2014). Bacteriological Analysis of sachet water sold in Akwa Metropolis, Nigeria. *International Journal of Agriculture and Biosciences*.

[19] Opara A. U., Nnodim J. (2014). Prevalence of Bacteria in bottled and sachet water sold in Owerri metropolis. *International Journal of Science Innovations and Discoveries*.

[20] Oyedeji O., Olutiola P. O., Moninuola M. A. (2010). Microbiological quality of packaged drinking water brands marketed in Ibadan metropolis and Ile-Ife city in South Western Nigeria. *African Journal of Microbiology Research*.

[21] Kim J., Li W., Philips B. L., Grey C. P. (2011). Storage effects on the quality of sachet water produced within Port Harcourt metropolis, Nigeria. *Journal of Biological Sciences*.

[22] Isikwue M. O., Chikezie A. (2014). Quality assessment of various sachet water brands marketed in Bauchi metropolis of Nigeria. *International Journal of Advances in Engineering and Technology*.

[23] Momba M. N. B., Mamba B. B., Mwabi J. K. (2012). Removal of waterborne bacteria from surface water and groundwater by cost-effective household water treatment systems (HWTS): A sustainable solution for improving water quality in rural communities of Africa. Environmental water and Earth science. *Water South Africa*.

[24] (2012). *APHA, AWWA and Water Environment Federation, Standard Methods for the Examination of Water and Waste Water*.

[25] ISO 16649-3 (2015). *Microbiology of the food chain, Horizontal method for the enumeration of beta-glucuronidase-positive Escherichia coli. Part 3: Detection and most probable number technique using 5-bromo-4-chloro-3-indolyl-ß-D-glucuronide*.

[26] ISO 6888-2 (2010). *Microbiology of food and animal feeding stuffs. Horizontal method for the enumeration of coagulase-positive Staphylococci (Staphylococcus aureus and other species). Part 2: Technique using rabbit plasma fibrinogen agar medium*.

[27] ISO 7937 (2016). *Microbiology of food and animal feeding stuffs. Horizontal method for the enumeration of Clostridium pafrigens, Colony-count technique*.

[28] ISO 9308-1 (2014). *Water quality -- Enumeration of Escherichia coli and coliform bacteria - Part 1: Membrane filtration method for waters with low bacterial background flora*.

[29] ISO 18743 (2015). *Microbiology of the food chain -- Detection of Trichinella larvae in meat by artificial digestion method*.

[30] ISO 18744 (2016). *Microbiology of the food chain. Detection and enumeration of Cryptosporidium and Giardia in fresh leafy green vegetables and berry fruits*.

[31] Close M., Dann R., Ball A., Pirie R., Savill M., Smith Z. (2008). Microbial groundwater quality and its health implications for a border-strip irrigated dairy farm catchment, South Island, New Zealand. *Journal of Water and Health*.

[32] Olaleye O. N., Ogunbajo A. H. (2015). Microbiological risk assessment of groundwater sources in Ikorodu- a peri-urban Lagos settlement. *Journal of Environmental Science and Water Resources*.

[33] Megha P. U., Kavya P., Murugan S., Harikumar P. S. (2015). Sanitation Mapping of Groundwater Contamination in a Rural Village of India. *Journal of Environmental Protection*.

[34] Venkatesan K., Balaji M., Victor K. (2014). Microbiological analysis of packaged drinking water sold in Chennai. *International Journal of Medical Science and Public Health*.

[35] Mahananda M. R., Mohanty B. P., Behera N. R. (2010). Physico-chemical analysis of surface and ground water of Bargarh district. *Orissa. India IJRRAS*.

[36] Manhokwe S., Matiashe I., Jombo T. Z. (2013). An analysis of the water quality of groundwater sources in selected high density areas in Gweru urban, Zimbabwe. *Journal of Environmental Science and Water Resources*.

[37] Al Sabahi E., Rahim S. A., Wan Zuhairi W. Y., Nozaily F. A., Alshaebi F. (2009). The characteristics of leachate and groundwater pollution at municipal solid waste landfill of Ibb City, Yemen. *American Journal of Environmental Sciences*.

[38] Razzolini M. T. P., Günther W. M. R., Peternella F. A. D. S. (2011). Quality of water sources used as drinking water in a Brazilian peri-urban area. *Brazilian Journal of Microbiology*.

[39] Lavanya V., Ravichandran S. (2013). Microbial contamination of drinking water at the source and household storage level in the peri-urban area of southern Chennai and its implication on health, India. *Journal of Public Health (Germany)*.

[40] Ahiablame L., Engel B., Venort T. (2012). Improving water supply systems for domestic uses in urban Togo: The case of a suburb in Lomé. *Water (Switzerland)*.

[41] Kirianki P. R., Othira J. O., Kiruki S. (2017). Analysis of Microbial Quality of Drinking Water in Njoro Sub-county, Kenya. *Journal of Environment Pollution and Human Health*.

[42] Kiruki S., Limo M., Njagi M., Okemo P. (2011). Bacteriological quality and diarrhoeagenic pathogens in River Njoro and Nakuru Municipal water, Kenya. *International Journal for Biotechnology and Molecular Biology Research*.

[43] Kumar Reddy D. H., Lee S. (2012). Water Pollution and Treatment Technologies. *Journal of Environmental & Analytical Toxicology*.

[44] Gu Q., Deng J., Wang K. (2014). Identification and assessment of potential water quality impact factors for drinking-water reservoirs. *International Journal of Environmental Research and Public Health*.

[45] Kelishadi R., Amin M. M., Haghdoost A. A., Gupta A. K., Tuhkanen T. A. (2013). Pollutants source control and health effects. *Journal of Environmental and Public Health*.

[46] Somani S. B., Ingole N. W. (2011). Alternative approach to chlorination for disinfection of drinking water an overview. *International Journal of Advanced Engineering Research and Studies*.

[47] McGuigan K. G., Conroy R. M., Mosler H.-J., du Preez M., Ubomba-Jaswa E., Fernandez-Ibañez P. (2012). Solar water disinfection (SODIS): A review from bench-top to roof-top. *Journal of Hazardous Materials*.

[48] KS EAS (2014). *Kenya Standard potable water specification*.

